# Abbreviated Breast MRI as a Supplement to Mammography in Family Risk History of Breast Cancer within the Croatian National Breast Screening Program

**DOI:** 10.3390/biomedicines12102357

**Published:** 2024-10-16

**Authors:** Andrea Šupe Parun, Boris Brkljačić, Gordana Ivanac, Vanja Tešić

**Affiliations:** 1 Croatian Institute for Public Health, 10 000 Zagreb, Croatia; 2 Breast Unit, Department of Diagnostic and Interventional Radiology, University Hospital Dubrava, 10 000 Zagreb, Croatia; boris@brkljacic.com (B.B.); gordana.augustan@gmail.com (G.I.); 3 School of Medicine, University of Zagreb, 10 000 Zagreb, Croatia; 4 Department of Epidemiology, Institute of Public Health “Dr. Andrija Štampar”, 10 000 Zagreb, Croatia; vanja.tesic@stampar.hr; 5 School of Medicine, University of Rijeka, 51 000 Rijeka, Croatia

**Keywords:** abbreviated breast MRI, mammography, breast cancer, breast cancer screening program

## Abstract

Objective: To evaluate the diagnostic performance of abbreviated breast MRI compared with mammography in women with a family history of breast cancer included in the Croatian National Breast Screening Program. Methods: 178 women with a family history of breast cancer aged 50 to 69 underwent abbreviated breast MRI and mammography. Radiological findings for each method were categorized according to the BI-RADS classification. The gold standard for assessing the diagnostic accuracy of breast MRI and mammography, in terms of suspicious BI-RADS 4 and BI-RADS 5 findings, was the histopathological diagnosis. Performance measures, including cancer detection rates, specificity, sensitivity, and positive and negative predictive values, were calculated for both imaging methods. Results: Twelve new cases of breast cancer were detected, with seven (58.3%) identified only by abbreviated breast MRI, four (33.3%) detected by both mammography and breast MRI, and one (8.3%) diagnosed only by mammography. Diagnostic accuracy parameters for abbreviated breast MRI were 91.67% sensitivity, 94.58% specificity, 55.0% positive predictive value (PPV), and 99.37% negative predictive value (NPV), while for mammography, the corresponding values were 41.67%, 96.39%, 45.46%, and 95.81%, respectively. Conclusions: Abbreviated breast MRI is a useful supplement to screening mammography in women with a family history of breast cancer. Considering the results of the conducted research, it is recommended to assess whether women with a family history of breast cancer have an increased risk and subsequently provide annual abbreviated breast MRI in addition to mammography for early detection of breast cancer.

## 1. Introduction

Breast cancer (BC) is an important public health problem, ranking as the second most commonly diagnosed cancer (11.6% of all cases across sexes) and the leading cause of cancer-related deaths among women [1]. However, in recent decades, mortality from BC has been declining, owing to increasingly advanced treatment and early detection through organized screening programs [2].

Early detection and diagnosis are essential for the treatment and prognosis of BC. With this goal in mind, the National Breast Cancer Early Detection Program was launched in the Republic of Croatia in 2006. As part of the program, all women aged 50–69 are invited to undergo mammography every two years, regardless of the risk for BC. However, women with a strong family history of BC, who are also part of the screening population, are more likely than others to develop BC. Between 15 and 20% of them have one or more first and/or second-degree relatives with BC [3]. To ensure that risk assessment is as accurate as possible, detailed data about family structure are needed, including the total number of relatives, their current age or age at death, and the age at the time of diagnosis [4,5]. The benefit for every woman would, thus, be to calculate the cancer risk and provide appropriate screening methods based on the level of risk.

Mammography is the primary method for the early detection of BC and a screening tool for asymptomatic women over 40, as it has been proven to reduce BC mortality by 22–33% [6]. It is accepted as the method of choice for women at an average risk of BC but not for those at high risk. For women at high risk of BC, the guidelines of the European Society of Breast Imaging (EUSOBI) and the American College of Radiology (ACR) recommend breast MRI as the screening method [7,8]. The high-risk group includes women with proven gene mutations and untested relatives in the first degree, followed by women with a calculated lifetime risk (LTR) > 20%, women who were irradiated in the chest area at a young age, as well as women with a personal history of breast cancer before the age of 50, or with a personal history of breast cancer and dense breasts from the time of diagnosis [8].

Numerous studies have shown that breast MRI may be a valuable supplement for screening high-risk women, with higher cancer detection rates than mammography alone [9,10,11,12,13,14,15,16,17]. They showed greater sensitivity of breast MRI compared to mammography, although biopsy rates and the number of additional cancers detected by breast MRI have varied from study to study.

Many authors have demonstrated that screening with breast MRI in high-risk women is characterized by high sensitivity and specificity, while some have also shown that the effectiveness can vary depending on the risk category [18,19]. Bick et al. concluded that risk assessments and inclusion criteria for breast MRI screening need to be adjusted to improve detection rates of new cancer cases [18]. Sippo et al. observed that the effectiveness of breast MRI is lower in women with a family history of BC compared to other groups with an increased risk and that a high-quality risk assessment strategy is very important for this group of women [19].

Many authors have demonstrated that breast MRI outperforms mammography in women genetically predisposed to breast cancer [10,11,12,14,16,17,18,19], but there are not many studies documented, to our knowledge, that have investigated the accuracy of screening using breast MRI in women with high familial risk and without known gene mutation. Their results were used for a meta-analysis by Phi et al., which included patients from several prospective studies [20].

The aim of our study was to evaluate the diagnostic performance of ABMRI compared with mammography in women with a family history of BC included in the National Breast Cancer Screening Program.

## 2. Materials and Methods

### 2.1. Study Design and Population

The study was conducted following ethical and medical principles and obtained approval from relevant institutional boards. All women participated voluntarily and provided informed consent before enrollment.

The study included 46,505 women aged 50 to 69 who underwent mammography as part of the 6th cycle of the National Breast Cancer Screening Program in the City of Zagreb. The data were collected from the National Breast Cancer Screening Program database by the Croatian Institute of Public Health. Upon arrival for the screening mammography, the women were asked to bring a filled-out survey that is an integral part of the invitation letter sent as part of the Croatian National Breast Screening Program. The survey contains questions about personal data but also data on family history. Their surveys, submitted upon arrival for mammography, were reviewed, and data on the family history of BC were considered for this research. All women with a family history of BC were included in the study according to the given criteria. The criteria for identification were as follows: one relative with BC in the first degree under the age of 40; two or more relatives with BC in the first degree, regardless of age; one relative with BC in the first degree and one relative with BC in the second degree, regardless of age; three or more relatives with BC in the second degree, regardless of age. First-degree relatives include a mother, father, daughter, son, sister, and brother, and second-degree relatives include a grandmother, grandfather, aunt, uncle, niece, nephew, half-sister, and half-brother [21]. After reviewing all 46,505 surveys, a total of 240 women with a family history of BC were identified, in which group a larger number of detected cancers was expected than in an average-risk population.

Women without a family history of BC, according to the given criteria, as well as women who underwent mammography or breast MRI within the last year, were excluded from the research.

All 240 women with a family history of BC were regularly invited for mammography as part of the latest 7th cycle of the screening program between March 2021 and February 2023. A total of 196 women responded to the invitation letter and, in addition to mammography, were also offered ABMRI. Out of the 196 women who underwent mammography, 11 declined ABMRI, and seven had contraindications for breast MRI. These contraindications included pacemakers, implanted magnetic devices, or severe obesity. In total, 178 women were included in the study.

### 2.2. Imaging Techniques and Findings

Mammograms were performed on the Selenia DIMENSIONS digital mammography unit (Hologic, Bedford, MA, USA) in two standard projections, followed by an invitation to ABMRI examinations.

ABMRI was performed within a month after the mammography. Following an abbreviated protocol, a breast MRI was performed on a 1.5T device (Avanto, Siemens, Erlangen, Germany). The protocol included the following sequences: transverse TIRM (turbo inversion recovery magnitude) and transverse 3D T1-weighted images with fat saturation, starting with a pre-contrast scan and followed by two dynamic post-contrast scans after intravenous administration of 0.1 mmol/kg of body weight of paramagnetic contrast agent gadoterate meglumine (Dotarem^®^, Guerbet, Paris, France) into the cubital vein, using an automatic injector at a speed of 2 mL/s, and finally the post-contrast administration of 20 mL of saline solution. During the examination, the women lay prone with their breasts positioned in the dedicated MRI coils, allowing them to hang freely within the coils to minimize motion artifacts.

Two experienced and highly trained radiologists were asked to read the images separately and independently, and disagreements were resolved by consensus. All mammograms and ABMRIs were coded according to the ACR Breast Imaging Reporting and Data System (BI-RADS) [22]. The overall assessment was performed according to six categories (0, incomplete or technically inadequate; 1, negative; 2, benign; 3, probably benign; 4, suspicious abnormality; 5, probably malignant). A final BI-RADS assessment category was provided for each finding for both imaging techniques. Only the findings categorized as BI-RADS 4 and BI-RADS 5 were considered suspicious findings. Consequently, women with BI-RADS 4 and BI-RADS 5 findings, either after mammography or ABMRI, were invited for further evaluation (clinical examination, breast ultrasound, and comparison with previous findings). According to these findings, it was decided which of the women classified as BI-RADS 4 and BI-RADS 5 would be scheduled for core needle biopsy.

### 2.3. Pathology

A histopathological analysis of the obtained material was performed, and the histological results were classified into B groups under the recommendations of the European Working Group for Breast Cancer Screening Pathology (EWGBSP). Data were collected on the number of invasive breast cancers, DCISs, and benign lesions, and a comparison was made of the number and types of breast lesions diagnosed by biopsy. The number of additional BC cases identified through BI-RADS 4 and BI-RADS 5 ABMRI findings, in comparison to mammography results, was also evaluated.

### 2.4. Statistical Methods

The Smirnov–Kolmogorov test was used to assess the normality of the distribution assessment. Quantitative data are presented through medians and interquartile ranges. Categorical data are presented through absolute frequencies, percentages, and 95% confidence intervals. Differences in quantitative values were analyzed with the Mann–Whitney U test. Differences in categorical variables were analyzed using Fisher’s exact test or Fisher–Freeman–Halton’s exact test in the case of contingency tables that were larger than the 2 × 2 format if there were less than eight subjects in each cell. Furthermore, the sensitivity and specificity, the area under the ROC curve (AUC), positive and negative likelihood ratios, positive and negative predictive values, as well as the test accuracy of individual diagnostic methods (mammography, breast MRI), were calculated in the detection of positive biopsy cancer findings.

All *p*-values under 0.05 were considered significant.

Program support IBM SPSS Statistics for Windows, version 29.0.1, was used in all statistical procedures.

## 3. Results

In total, 178 mammograms and ABMRIs were performed at the Department of Diagnostic and Interventional Radiology of University Hospital Center Zagreb between March 2021 and February 2023. The women’s ages ranged from 50 to 69 years, with the average age being 57.3 ± 6.0 years. The age range of their relatives with BC was 26 to 87 years, with an average age of 45.2 ± 11.4 years.

Women (N = 178) were classified into four different categories depending on the type and age of the relatives. Almost half of the women (49.4%) had a relative with BC under the age of 40 in the first degree, while the smallest proportion (8.4%) had three relatives with BC in the second degree, regardless of age. The largest share of women (54.5%) listed their mother as a relative, followed by their sister (36.0%) and aunt (33.1%). The two smallest shares were associated with the daughter (5.1%) and niece (1.7%) as the relatives.

In mammography, the finding of category BI-RADS 2 was the most common in 140 women (78.7%), while categories BI-RADS 1 and BI-RADS 5 were the least represented, with a share of 2.2%. At ABMRI, the most common finding was BI-RADS 2 in 124 women (69.7%), and the least common finding in two women (1.1%) was BI-RADS 1. In terms of suspicious findings, 11 (6.2%) BI-RADS 4 and BI-RADS 5 categories were classified by mammography, and 20 (11.2%) were classified by ABMRI. The classification of mammography and ABMRI findings is summarized in Table 1.

A total of 17 biopsies were performed, of which five (29.4%) were negative, while nine (52.9%) were positive for invasive BC, and three (17.6%) for DCIS. Four women with suspicious findings detected by ABMRI (BI-RADS 4), including one BI-RADS 4 also detected by mammography, had no additional treatment performed. In the case of one woman, the reason was a lack of consent for additional treatment, while the remaining three decided to undergo additional treatment in other health institutions. Also, four women who had a suspicious result detected by mammography (BI-RADS 4) but a benign result according to ABMRI (BI-RADS 2) after additional treatment that included a clinical examination and ultrasound were not referred for a biopsy but were recommended to report for a check-up in six months. The biopsy findings are summarized in Table 1.

**Figure 1:**
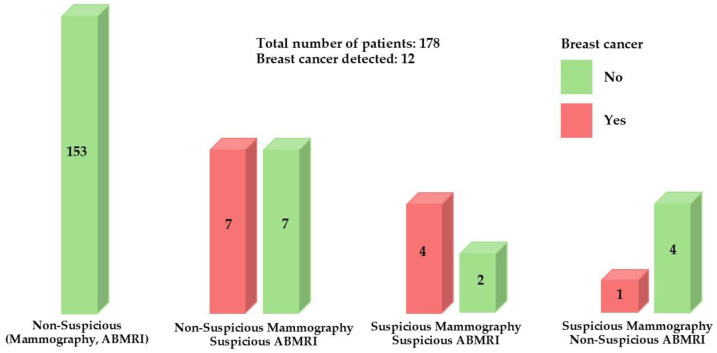
Number of biopsy-confirmed breast cancer cases according to suspicious mammography and ABMRI.

A statistically significant difference (*p* < 0.001) was recorded in the findings of imaging methods between women whose BC was confirmed by biopsy and those whose was not. One woman with BC had a positive mammography and a negative ABMRI, seven women with BC had a negative mammography and a positive ABMRI, and four women with BC had a positive mammography and positive ABMRI (Figure 1). According to these data, we can confirm that, with the introduction of ABMRI during screening, seven cases of BC were discovered that would otherwise have remained undetected (since they were negative on mammography). Differences in mammography and breast MRI findings are summarized in Table 2.

**Figure 2:**
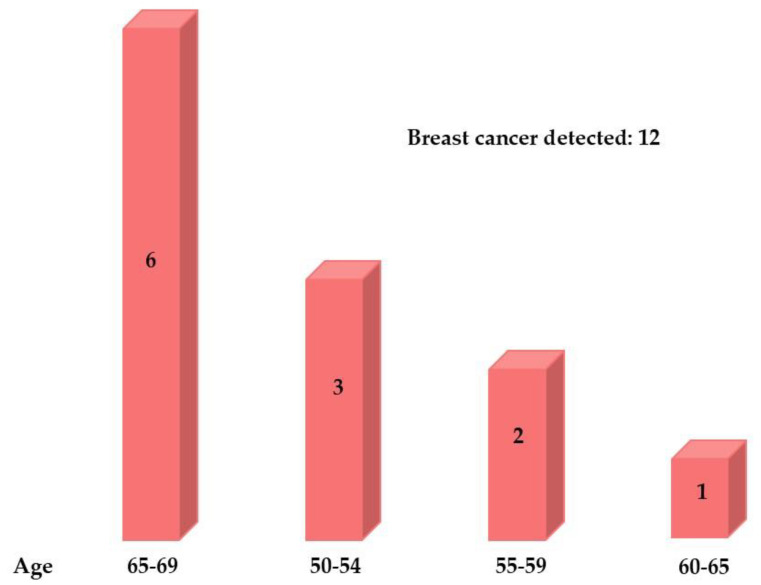
Distribution of breast cancer across age groups.

The largest number of BC cases, six (50%), to be exact, were detected in the oldest 65–69 age group, accounting for one-half of the total detected BC cases (Figure 2).

The overall diagnostic performance of ABMRI was superior to that of mammography. ABMRI, in particular, showed better sensitivity (91.7% versus 41.7%) than mammography, while specificity was slightly lower but not statistically significant (94.6% versus 96.4%).

The diagnostic value of mammography alone in BI-RADS suspicious findings was characterized by 41.7% sensitivity (95% CI 15.2–72.3), 96.4% specificity (95% CI 92.3–98.7), 45.5% PPV (95% CI 22.9–70.1), and 95.8% NPV (95% CI 93.4–97.4). Data on the overall diagnostic performance of mammography are summarized in Table 3.

The diagnostic value of ABMRI in BI-RADS suspicious findings was associated with 91.7% sensitivity (95% CI 61.5–99.8), 94.6% specificity (95% CI 90.0–97.5), 55.0% PPV (95% CI 38.8–70.2), and 99.4% NPV (95% CI 96.0–99.9). Data on the overall diagnostic performance of ABMRI are summarized in Table 4.

By combining mammography and ABMRI in BI-RADS suspicious findings, the resulting sensitivity was 100% (95% CI 73.5–100.0), as the combination of both methods covered all cases of BC, 12, to be exact, while specificity was still very high at 92.2% (95% CI 87.0–95.8). Data on the overall diagnostic performance of the combination of mammography and ABMRI are summarized in Table 5.

## 4. Discussion

Breast MRI, in addition to mammography, is recommended for women running a 20% or greater LTR for developing BC, including women with a strong family history of BC [8,23].

It is now established that breast MRI has a very high sensitivity in BC detection, but also certain limitations.

It was demonstrated that ABMRI has a similar diagnostic performance as breast MRI according to a regular full protocol, with some benefits to breast MRI according to full protocol, including simpler application, shorter examination times, report reading, and lower costs [24,25,26,27].

Considering the well-known limits of breast MRI according to a regular full diagnostic protocol, it was decided to propose an abbreviated protocol for women with a family history of BC.

Our findings show that ABMRI in women with a family history of BC is more sensitive than mammography. ABMRI was a very effective method in detecting cancer, as out of the 12 cases of BC, 11 (92%) were detected by ABMRI. Of the twelve BC cases detected in total, seven (58.3%) were detected only by ABMRI, one (8.3%) was detected only by mammography, and four (33.3%) were detected by a combination of ABMRI and mammography. We can confirm that seven additional malignant lesions were identified at ABMRI. Thus, achieving a higher detection rate can lead to earlier staging, better prognosis due to early intervention, improved quality of life, and more personalized treatment planning.

The results of our study have indicated that almost all considered parameters had superior values at ABMRI in comparison to mammography alone.

In particular, ABMRI reached a 91.7% sensitivity, which is significantly higher compared to mammography, whose sensitivity was only 41.7%.

According to most literature data, the sensitivity of breast MRI ranges from 80 to 100% and is comparable with our results [28,29,30,31].

Our findings have also indicated excellent specificity for ABMRI (94.6%), slightly lower than mammography (96.4%), but without statistical significance. According to literature data, the specificity of breast MRI ranges from 72 to 98% [32,33,34].

Based on the obtained sensitivity and specificity of ABMRI, our results are at the upper end of published studies and are very similar to the results published by Kuhl et al. in women with familial risk of BC, although the study also included women with a genetic predisposition to BC [11].

Additionally, our results are consistent with the findings of the biggest studies conducted on the population of women with strong familial BC risk but without a known gene mutation, which have also reported higher sensitivity of breast MRI compared to mammography [20]. Moreover, in the study by Phi et al., mammography screening had a sensitivity of 55% and a specificity of 94%, while screening with MRI alone had a sensitivity of 89% and a specificity of 83%, which is very similar to the results obtained in our research.

In the study by Kuhl et al., when ABMRI was applied in cases of a slight-to-moderate increased risk, the reported diagnostic performance measures (sensitivity, specificity, and PPV) were similar to ours despite an even lower PPV [24].

Our performance measures have demonstrated that the diagnostic accuracy of ABMRI was highly effective in terms of sensitivity (91.7%), specificity (94.6%), and negative predictive values (99.4%), which supports the reliability of the method in detecting BC, the low rate of false-negative results, and the confidence that a negative breast MRI finding most likely eliminates the presence of cancer.

However, the study results report a positive predictive value of 55.0% for ABMRI. While the sensitivity of ABMRI is high, the relatively low PPV might imply a substantial number of false-positives. This can lead to several potential risks and impacts on patient anxiety, unnecessary additional procedures, and healthcare costs. False-positive findings may cause patients to lose confidence in the accuracy of breast MRI screening and could potentially lead to lower screening adherence, where patients might avoid future screenings out of fear of experiencing another false-positive or undergoing more unnecessary procedures. However, proper counseling and clear communication about the possibility of false-positives can help alleviate some of the anxiety. Also, minimizing false-positives through further improving imaging techniques or combining ABMRI with other screening methods could help improve diagnostic accuracy and reduce the psychological and clinical burden on patients. Our study thus combined mammography and ABMRI with ultrasound before referring women for more invasive diagnostics.

For patients with a family history of BC, the higher sensitivity of breast MRI often outweighs the potential increase in false-positives, as earlier detection is a priority. Mammography, with its higher specificity, may still be preferred in lower-risk women, where the likelihood of false-positives may lead to unnecessary anxiety and medical procedures. Therefore, in high-risk women, breast MRI is frequently recommended despite the possibility of additional follow-up tests because early detection is often crucial. It is known from the works of other authors that MRI-detected cancers are smaller than tumors detected by conventional mammography and that biologically aggressive cancers are more likely to be detected by MRI [35,36].

In our study, histopathological diagnosis confirmed the detected BC. However, there are several potential limitations associated with using histopathology as the gold standard in this context, which could affect the reported diagnostic accuracy of ABMRI. These limitations include a risk that the biopsy may not capture all areas of a heterogeneous tumor or may miss malignant regions if the sampling is not comprehensive; the interpretation of histopathology can be subjective, depending on the expertise and experience of the pathologist; BC can evolve or change between the ABMRI and biopsy. Non-compliance between what ABMRI identifies and what histopathology confirms may result in the ABMRI appearing less sensitive or specific than it is. However, although biopsy may have limitations, it is essential in establishing a cancer diagnosis.

Chiarelli et al., in a study in 2020, which included 8782 women, found that among high-risk women aged 50 to 69, early cancer detection was most effective when mammography was included in an annual breast MRI, while for younger women between the ages of 30 and 39, an annual breast MRI was sufficient as a method, especially for BRCA1 and BRCA2 mutation carriers [16]. These data support the fact that women between the ages of 50 and 69, regardless of the increased risk, should not be excluded from regular mammographic examinations but instead offered an additional annual breast MRI examination. Screening with mammography and breast MRI increases the sensitivity to 100% and allows for the detection of BC among women with high risk.

To optimize the use of ABMRI, it is important to assess the risk of a woman developing BC. For this purpose, several different models have been developed to calculate LTR [37]. Some of the well-known models used in practice for risk assessment are Gail, Claus, Tyrer–Cuzick, BOADICEA, and the Claus Extended Formula, along with genetic testing for markers like BRCA1/BRCA2. They should be integrated into routine screening programs, especially for women with a family history of BC. Personalized risk assessments based on these tools can lead to a decision on who qualifies for screening with ABMRI, while unnecessary interventions are minimized for average-risk women. This approach will allow for more precise, efficient, and woman-centered breast cancer screening.

The potential research directions for exploring the role of ABMRI for the Croatian National Breast Screening Program can focus on refining risk stratification tools to identify the most appropriate populations for ABMRI screening. While ABMRI is particularly useful for those with genetic mutations (like BRCA1/BRCA2), more precise guidelines are needed to balance out the cost-effectiveness of ABMRI with its benefits for family-risk women without known genetic mutations. Studies could explore the connection of the risk assessment models (e.g., the Gail model, Tyrer–Cuzick, and Claus) with ABMRI so that only those at sufficiently high risk are screened with ABMRI.

When it comes to a large-scale application of new expensive tests, aside from their efficiency, their cost-effectiveness should also be considered. Screening programs are required to provide high efficiency and favorable economic outcomes.

Despite a significant diagnostic value, high costs, among other reasons, have prevented breast MRI from taking a prominent role in screening. Recently conducted economic analyses indicate an added value in the application of breast MRI for high-risk women and its superiority over other methods, which could be the base for more frequent use of breast MRI as a screening technique [38,39]. ABMRI, as a supplement to mammography, can increase healthcare costs, but this could be justified by several long-term benefits, such as improving the detection of BC, detection of BC at an earlier stage, reducing mortality, and potentially less aggressive treatment. By targeting high-risk women and improving screening protocols, healthcare systems can balance out the cost-effectiveness and diagnostic benefits of ABMRI. Our results also suggest that it is feasible to offer breast MRI screening to women with a family history of BC, thereby detecting additional cases of BC, potentially at an earlier stage than by mammography [38,39].

Our study has encountered some limitations, though: firstly, there was a relatively small number of women who were invited for ABMRI; secondly, a selection bias may be present as our sample included the data on family history of BC based on a survey filled out by the participating women themselves; finally, the decision to categorize only BI-RADS 4 and BI-RADS 5 findings as positive may have impacted the obtained results.

In conclusion, our results have demonstrated that ABMRI has much better diagnostic performance, compared to mammography, in women with a family history of BC within the Croatian National Breast Cancer Screening Program. The integration of ABMRI could lead to more personalized screening strategies. Considering that not many women have been assessed for a BC risk, participation in a screening program can be an opportunity for this. Women at sufficiently high risk can undergo ABMRI screenings, while those at average risk continue with standard mammography, optimizing resources and improving outcomes. These changes would enhance early detection and likely improve survival rates in this vulnerable population.

## Figures and Tables

**Table 1:** Classification of mammography and ABMRI findings and biopsy results.

		N	%	95% CI	
Mammography BI-RADS	0	19	10.7%	6.8%	15.8%
	1	2	1.1%	0.2%	3.6%
	2	140	78.7%	72.2%	84.2%
	3	6	3.4%	1.4%	6.8%
	4	9	5.1%	2.5%	9.0%
	5	2	1.1%	0.2%	3.6%
ABMRI BI-RADS	0	3	1.7%	0.5%	4.4%
	1	2	1.1%	0.2%	3.6%
	2	124	69.7%	62.6%	76.1%
	3	29	16.3%	11.4%	22.2%
	4	14	7.9%	4.6%	12.5%
	5	6	3.4%	1.4%	6.8%
Biopsy	Without biopsy	161	90.4%	85.5%	94.1%
	B2	5	2.8%	1.1%	6.0%
	B5a	3	1.7%	0.5%	4.4%
	B5b	9	5.1%	2.5%	9.0%
Biopsy *	Invasive cancer	9	52.9%	30.3%	74.6%
	DCIS	3	17.6%	5.2%	40.0%
	Negative	5	29.4%	12.2%	53.0%
Mammography BI-RADS	Others	167	93.8%	89.6%	96.7%
	Suspicious (BI-RADS 4/5)	11	6.2%	3.3%	10.4%
ABMRI BI-RADS	Others	158	88.8%	83.5%	92.8%
	Suspicious (BI-RADS 4/5)	20	11.2%	7.2%	16.5%

Biopsy * = number of biopsies performed, N = number of findings, CI = confidence interval.

**Table 2:** Differences in mammography and ABMRI findings.

		Breast Cancer				*p*
		No		Yes		
		N	%	N	%	
Mammography + ABMRI	Non-suspicious mammography and non-suspicious ABMRI	153	92.2%	0	0.0%	<0.001
	Suspicious mammography and non-suspicious ABMRI	4	2.4%	1	8.3%	
	Non-suspicious mammography and suspicious ABMRI	7	4.2%	7	58.3%	
	Suspicious mammography and suspicious ABMRI	2	1.2%	4	33.3%	

N = number of findings, *p* = *p*-value.

**Table 3:** Diagnostic performance of mammography in BI-RADS positive findings.

	Value	95% CI
Sensitivity	41.67%	15.165–72.333%
Specificity	96.39%	92.299–98.662%
AUC	0.69	0.617–0.757
Positive odds ratio	11.528	4.107–32.359
Negative odds ratio	0.605	0.375–0.977
PPV	45.46%	22.891–70.053%
NPV	95.81%	93.402–97.362%
Accuracy	92.70%	87.835–96.054%

AUC = area under the curve, PPV = positive predictive value, NPV = negative predictive value, CI = confidence interval.

**Table 4:** Diagnostic performance of ABMRI in BI-RADS positive findings.

	Value	95% CI
Sensitivity	91.67%	61.520–99.789%
Specificity	94.58%	89.958–97.491%
AUC	0.931	0.884–0.964
Positive odds ratio	16.907	8.757–32.643
Negative odds ratio	0.088	0.013–0.576
PPV	55.00%	38.765–70.236%
NPV	99.37%	96.005–99.903%
Accuracy	94.38%	89.911–97.273%

AUC = area under the curve, PPV = positive predictive value, NPV = negative predictive value, CI = confidence interval.

**Table 5:** Diagnostic performance of the combination of mammography and ABMRI in BI-RADS positive findings.

	Value	95% CI
Sensitivity	100.00%	73.535–100.000%
Specificity	92.17%	86.981–95.764%
AUC	0.961	0.921–0.984
Positive odds ratio	12.769	7.577–21.519
Negative odds ratio	0	
PPV	48.00%	35.390–60.870%
NPV	100.00%	97.618–100.000%
Accuracy	92.70%	87.835–96.054%

AUC = area under the curve, PPV = positive predictive value, NPV = negative predictive value, CI = confidence interval.

## Data Availability

Data available on request.
